# Isolated downregulation of HCN2 in ventricles of rats with streptozotocin-induced diabetic cardiomyopathy

**DOI:** 10.1186/s12872-021-01929-3

**Published:** 2021-03-02

**Authors:** Katarina Hadova, Eva Kralova, Gabriel Doka, Lenka Bies Pivackova, Zuzana Kmecova, Peter Krenek, Jan Klimas

**Affiliations:** Department of Pharmacology and Toxicology, Faculty of Pharmacy, Comenius University in Bratislava, Odbojarov 10, 832 32 Bratislava, Slovakia

**Keywords:** HCN channels, Arrhythmias, Diabetic cardiomyopathy, Heart, Electrophysiology, Rat

## Abstract

**Background:**

In spite of disrupted repolarization of diabetic heart, some studies report less tendency of diabetic heart to develop ventricular arrhythmias suggesting effective compensatory mechanism. We hypothesized that myocardial alterations in HCN2 and HCN4 channels occur under hyperglycaemia.

**Methods:**

Diabetes was induced in rats using a single injection of streptozotocin (STZ; 55 mg/kg body weight, i.p.). Basal ECG was measured. Expression of mRNA for HCN channels, potassium channels and microRNA 1 and 133a were measured in ventricular tissues. Protein expression of HCN2 channel isoform was assessed in five different regions of the heart by western blotting. Differentiated H9c2 cell line was used to examine HCN channels expression under hyperglycaemia in vitro.

**Results:**

Six weeks after STZ administration, heart rate was reduced, QRS complex duration, QT interval and T-wave were prolonged in diabetic rats compared to controls. mRNA and protein expressions of HCN2 decreased exclusively in the ventricles of diabetic rats. HCN2 expression levels in atria of STZ rats and H9c2 cells treated with excess of glucose were not changed. MicroRNA levels were stable in STZ rat hearts. We found significantly decreased mRNA levels of several potassium channels participating in repolarization, namely Kcnd2 (*I*_to1_), Kcnh2 (*I*_Kr_), Kcnq1 (*I*_Ks_) and Kcnj11 (*I*_KATP_).

**Conclusions:**

This result together with downregulated HCN2 channels suggest that HCN channels might be an integral part of ventricular electric remodelling and might play a role in cardiac repolarization projected in altered arrhythmogenic profile of diabetic heart.

## Background

Diabetes mellitus (DM) is considered to be a strong risk factor for sudden cardiac death [1]. Diabetic heart exhibits pro-arrhythmogenic electrocardiographic abnormalities such as abnormal repolarization [2] mostly related to potassium channels alterations [3]. Therefore, the link between DM and related risk of arrhythmias should be straightforward. Intriguingly, there are some experimental studies [4, 5] as well as clinical trials [6, 7] suggesting less tendency of the diabetic heart to develop ventricular arrhythmias in certain conditions. Several explanations of this peculiar phenomenon were suggested [4, 5], but its exact molecular mechanism is still not elucidated.

Cardiac HCN channels are of particular importance for heart rhythm. This has been documented by HCN2- or HCN4-deficient mice which exhibit sinoatrial dysfunction [8–10] and also by development of bradycardic HCN channel blocking agent ivabradine [11]. On the other hand, in ventricular myocardium, their overexpression contributes to cardiac pro-arrhythmogenic potential [12]. All four isoforms of HCN channels are expressed in cardiac tissue but they manifest a species-dependent regional specific distribution [10]. HCN2 and HCN4 isoforms are predominant ventricular HCN transcripts and together represent more than 90% of the ventricular HCN channels [13]. However, in healthy atrial and ventricular cardiomyocytes which do not display spontaneous activity [10], HCN channels are barely expressed when compared to their stable expression in pacemaker cells [14]..An enhanced expression of myocardial HCN channels contributes to increased pacemaker current (*I*_f_) which relates to ventricular and atrial arrhythmias in failing hearts [15]. Moreover, HCN channels, when overexpressed, were reported to prolong the repolarization of ventricular action potential and thereby increase the arrhythmogenic potential [14]. HCN channels are supposed to functionally antagonize K^+^ currents during late repolarization thanks to slow deactivation kinetics. Therefore, their upregulation in hypertrophic heart relates to prolonged QT interval and the increased arrhythmogenic potential [13]. Furthermore, *I*_f_ augmentation induces a diastolic influx of Na^+^ cations leading to increased intracellular Ca^2+^ due to the Na^+^/Ca^2+^ exchanger shift towards 'reverse mode' resulting in increased arrhythmogenicity [16]. The overexpression of myocardial HCN occurs in various conditions, including cardiac hypertrophy [17], acute myocardial infarction [18] and heart failure [15] while the reduction of myocardial HCN is rather related to atria and to impaired sinus rhythm [19, 20].

On the other hand, blockade of cardiac HCN channels by ivabradine is clinically used to treat systolic heart failure and chronic angina indicating beneficial effect of attenuation of HCN channels activity in the heart disease [11]. Furthermore, cardiac HCN channels blockade was shown to reduce lethal arrhythmias in dilated cardiomyopathy [12]. Interestingly, although clinical studies reported efficacy and safety of ivabradine in diabetic patients [21], cardiac effects of ivabradine have been reported to be weaker in rats with streptozotocin (STZ)-induced diabetic heart damage [22]. Recently, reduced expression of HCN channels in the sinoatrial node of streptozotocin (STZ)-induced diabetic rats was found [20]. Therefore, we hypothesize that altered cardiac electrogenesis in diabetes might be affected by changes in cardiac HCN channel expression.

## Methods

### Animals

For the purpose of the experiment, 12 weeks old male Wistar rats (Department of Toxicology and Laboratory Animal Breeding, Dobra Voda, Slovak Republic) were used. The rats were housed in our facility in air-conditioned quarters (21 ± 1 °C). The relative humidity was 40–70% and a photoperiod was 12-h. The rats had free access to water and normal rodent chow. The use of experimental animals, including experimental procedures were approved by the State Veterinary and Food Administration of the Slovak Republic and by the Ethics Committee of the Faculty of Pharmacy, Comenius University, Bratislava. The investigation follows the Guide for the Care and Use of Laboratory Animals: Eighth Edition (2010) published by the US Committee for the Update of the Guide for the Care and Use of Laboratory Animals; National Research Council, the EU adopted Directive 2010/63/EU of the European Parliament and of the Council on the protection of animals used for experimental and other scientific purposes and the Slovak law regulating animal experiments.

### Treatment

Diabetes mellitus heart damage was induced with streptozotocin (STZ) as we previously described [2]. The animals were randomized into control (CON, n = 10) and diabetic (STZ, n = 10) group. STZ was injected by a single i.p. injection of streptozotocin in a dose of 55 mg/kg to induce type 1 diabetes mellitus. This model is known for its peculiar protection against triggered dysrhythmias [4, 5]. Streptozotocin was dissolved in 0.1 M citrate buffer (pH 4.5). Control rats were i.p. injected with citrate buffer only. After 6 weeks random blood glucose levels were measured by a glucometer Accutrend® Plus (Roche®, Switzerland) from the tail vein blood samples and afterwards the rats were sacrificed by exsanguination in tribromethanol anaesthesia (250 mg/kg i.p. Sigma Aldrich, Saint Louis, MO, USA). The samples of left and right ventricular free wall and left and right atria and intraventricular septum were excised, quickly frozen in liquid nitrogen and stored at − 80 °C until analysed.

### Basal ECG measurements

Rats were anaesthetized with 2.5% solution of tribromethanol (250 mg/kg i.p. Sigma Aldrich, Saint Louis, MO, USA) and standard 12-lead electrocardiography with needle electrodes was performed as previously described [2]. 1.5 cm from the xyphoid process on the sternum was the centre of the chest electrodes. ECG parameters were estimated using a SEIVA EKG PRAKTIK (Prague, Czech Republic). PQ interval was determined from the onset of P wave to the onset of QRS complex. In the same manner QT duration was measured. The beginning was determined by the onset of QRS complex and the end of T wave. The measurements were evaluated semimanually in the II. lead. Three consecutive beats were evaluated, and the arithmetic means of all parameters were obtained. Duration of QT was corrected [23] as QTc (in ms) = QT/(RR/150)^2^.

### Cell culture

To exclude the effect of altered cardiac electrogenesis on HCN expression and to answer the importance of maturation of cardiac cells in observed hyperglycaemia-related alterations of Hcn2 expression, we used in vitro culture of H9c2 (2–1) (RRID:CVCL_0286) myogenic cell line derived from embryonic rat heart ventricle [24]. These cells do not have apparent endogenous pacemaker currents and can serve as reasonable models for investigation of HCN expression [25]. The H9c2 cell line purchased from America Tissue Type Collection (ATCC®, Manassas, VA; catalogue #CRL-1446) was cultured in DMEM medium (ATCC® Manassas, VA; catalogue #30-2002™) containing 25 mM glucose supplemented with 10% fetal bovine serum (FBS; ATCC® Manassas, VA, catalogue #30-2020™), 100 U/ml penicillin and 100 μg/ml streptomycin in 75 cm^2^ tissue culture flasks at 37 °C in a humidified atmosphere of 5% CO_2_. Cell medium was replaced every 2–3 days, and cells were sub-cultured when reaching 60–70% confluence in order to prevent the loss of the differentiation potential. Cardiac differentiation of H9c2 cells was initiated by decreasing the percentage of serum (1% FBS) and glucose (5.5 mM) in the media. H9c2 cells were plated at a density of 4 500 cells/cm^2^ in 75 cm^2^ tissue culture flasks and were fed every 2 days with fresh medium. Gene expression of cardiac troponin T (*Tnnt2*) was used as a marker of differentiation. After 7 days of differentiation cells were plated at a density of 4 500 cells/cm^2^ in 6-well plate and were divided into several groups: cells treated with medium containing 1% FBS with low glucose (LG-DIF; 5.5 mM), high glucose (HG-DIF; 33 mM), or low glucose (5.5 mM) plus mannitol (M-DIF; 27.5 mM) to exclude the effect of distinct osmotic conditions between groups. The cells were maintained under these experimental conditions for 48 h with the change of fresh medium after 24 h. At the end of the experiment, the cells were lysed using TRI reagent® (Sigma-Aldrich, St. Louis, MO, USA) and RNA isolation was carried out. Additionally, the sample of non-differentiated (NON-DIF; n = 2) cells was harvested before the differentiation was initiated and cardiac troponin T expression was compared with the samples harvested after the whole course of experiment. In addition, the cells treated in the same manner were fixed with 4% formaldehyde and labelled with Wheat Germ Agglutinin (WGA) Alexa Fluor 488 Conjugate according to manufacturer´s instructions and visualized using fluorescence microscopy to assess the morphology of the cells before and after differentiation.

### RNA isolation and quantitative RT-PCR

Left ventricle samples were snap-frozen in liquid nitrogen, stored at − 80 °C until RNA extraction by acid phenol–guanidinium thiocyanate–chloroform extraction (TRI Reagent®, Sigma- Aldrich, St. Louis, MO, USA) according to the manufacturer´s instructions. Total RNA of both left ventricle samples and H9c2 cells were isolated and treated according to same protocol. For RNA quality control electrophoresis in 2% agarose gel (Agarose, Sigma- Aldrich, USA) was used. Intact RNA was reverse transcribed using High capacity cDNA Reverse Transcription Kit with RNAse inhibitors (Applied Biosystems, Grand Island, NY, USA). For quantitative real-time PCR (RT-qPCR) SYBR™ Select Master Mix (Thermo Fisher Scientific, USA) was used. The analysis was performed on StepOnePlus™ Real-Time PCR System (Thermo Fisher Scientific, USA). We tested mRNA expressions of Hcn2 and Hcn4 genes, other cardiac ion channels genes and genes related to cardiac damage (shown in Table 1) [26, 27]. Cardiac damage was assumed on the basis of measured shift in Myh6 and Myh7 gene expression [28, 29]. The primers used for analysis were designed using Primer-BLAST [30]. Pfaffl method [31] was used to calculate the relative expression. Results were normalized to the geometric mean of two most suitable reference genes for each sample type [32]. We used hypoxanthine phosphoribosyltransferase 1 (Hprt1) and beta-2-microglobulin (B2m) for left ventricle samples and B2m and succinate dehydrogenase complex flavoprotein subunit A (Sdha) for H9c2 cells. Calculated normalized quantities were calibrated to appropriate control groups.

**Table 1:** Primer sequences for RT-qPCR and mature sequences of rat cardiac microRNAs and control U6 snRNA

Primer sequences for RT-qPCR			
Gene	RefSeq accession number	Primer sequence (5′–3′)	PCR product length (bp)
B2m	NM_012512.1	Forward: ATGGAGCTCTGAATCATCTGG Reverse: AGAAGATGGTGTGCTCATTGC	105
Hcn2	NM_053684.1	Forward: CTGACACCTACTGTCGCCTC Reverse: TCTTCTTGCCTATGCGGTCC	129
Hcn4	NM_021658.1	Forward: AGTATCCCATGATGCGCAGG Reverse: TTCTTCTTGCCTATGCGGTCC	70
Hprt1	NM_012583.2	Forward: CAGCTTCCTCCTCAGACCGCTTT Reverse: TCACTAATCACGACGCTGGGACTG	150
Kcnd2	NM_031730.2	Forward: CCTGGAGAAAACCACGAACC Reverse: TGCTGGTGACTCCTTGTTGG	131
Kcnh2	NM_053949.1	Forward: GACCTGCTTACTGCCCTCTAC Reverse: GACGTGCATACAGGTTCAGAG	124
Kcnj11	NM_031358.3	Forward: CACACAGCCACGACAGGATA Reverse: CGTCTGAACGGGACCATCAA	117
Kcnj2	NM_017296.1	Forward: AACCGCTACAGCATCGTCTC Reverse: GCACTGTTGTCGGGTATGGA	111
Kcnq1	NM_032073.1	Forward: CTGGTCTGCCTCATCTTCAG Reverse: TCTGTCCCAAAGAACACCAC	110
Myh6	NM_017239.2	Forward: GCCCTTTGACATCCGCACAGAGT Reverse: TCTGCTGCATCACCTGGTCCTCC	152
Myh7	NM_017240.1	Forward: GCGGACATTGCCGAGTCCCAG Reverse: GCTCCAGGTCTCAGGGCTTCACA	133
Ryr2	NM_001191043.1; NM_032078.2	Forward: ACTGCTGGGCTACGGCTAC Reverse: CTGAAGATGCGGAACCTCTC	99
Sdha	NM_130428.1	Forward: GTGTTGCTGTGTCGCTGATC Reverse: AATGACACCACGGCACTCC	142
Slc2a1 (Glut1)	NM_138827.1	Forward: ATTCTCCGTTTCACAGCCCG Reverse: CTCCTCAATTACCTTCTGGGGG	176
Slc2a4 (Glut4)	NM_012751.1	Forward: GACCCGCCCTTTGCACACCA Reverse: TCACTCGCTGCTGAGGGGGT	174
Tnnt2	NM_012676.1	Forward: GACAGGATCGAAAAGCGTCG Reverse: AGCCTTCCTCCTGTTCTCCT	132
Tnni3	NM_017144.2	Forward: GAGCTTCAGGACCTATGCCG Reverse: AACTTGCCACGCAGGTCATA	143

Mature sequences of rat cardiac microRNAs			
microRNA name	Nomenclature of mature form	Sequence	Assay ID
miR-1	rno-miR-1-3p	5′-UGGA AUG UAA AGA AGU GUG UAU-3′	002064
miR-133a	rno-miR-133a-3p	5′-UUUG GUC CCC UUC AAC CAG CUG-3′	002246
U6	U6 snRNA	5′ GTGCTCGCTTCGGCAGCACATATAC TAAAATTGGAACGATACAGAGAAGAT TAGCATGGCCCCTGCGCAAGGATGAC ACGCAAATTCGTGAAGCGTTCCATATT TT-3′	001973

Seed microRNAs sequences are underlined, source mirBase.org

B2m: β2-Microglobulin; Hcn2: hyperpolarization activated cyclic nucleotide gated potassium and sodium channel 2; Hcn4: hyperpolarization activated cyclic nucleotide gated potassium and sodium channel 4; Hprt1: hypoxanthine phosphoribosyltransferase 1; Kcnd2: potassium voltage-gated channel subfamily D member 2; Kcnh2: potassium voltage-gated channel subfamily H member 2; Kcnj11: potassium inwardly rectifying channel subfamily J member 11; Kcnj2: potassium inwardly rectifying channel subfamily J member 2; Kcnq1: potassium voltage-gated channel subfamily Q member 1; Myh6: myosin heavy chain 6 (α‐myosin heavy chain); Myh7: myosin heavy chain 7 (β‐myosin heavy chain); Ryr2: ryanodine receptor; 2 Sdha: succinate dehydrogenase complex flavoprotein subunit A; Slc2a1: solute carrier family 2 member 1 (alias Glut1); Slc2a4: solute carrier family 2 member 4 (alias Glut4); Tnnt2: troponin T2, cardiac type; Tnni3: troponin I3, cardiac type;

### microRNA expression measurement

The isolated left ventricle total RNA samples were also used to determine microRNA expression. Reverse transcription for microRNA assay was performed using TaqMan MicroRNA Reverse Transcription Kit (Applied Biosystems, USA) in a multiplex assay of analysed microRNAs (miR-1-3p, miR-133a-3p; Table 1) and endogenous reference (U6 small nuclear RNA; Table 1) according to the manufacturer’s instructions. For qPCR reactions with primers from individual TaqMan microRNA Assays (Applied Biosystems, USA; Table 1) TaqMan 2 × Universal PCR Master Mix (Applied Biosystems, USA) was used. MicroRNAs qPCR reaction and final calculations were performed in the similar manner as in mRNA assay.

### SDS-PAGE and western blotting

The samples of left and right ventricular free walls, ventricular septum and left and right atrium were snap-frozen in liquid nitrogen and homogenized as described previously [33]. Samples were subjected to sodium dodecyl sulphate- polyacrylamide gel electrophoresis on a 9% gel and transferred to polyvinylidene fluoride membrane (Immobilon P®, Millipore Corporation, USA). Membranes were blocked with 5% non-fat dried milk in TBST and subsequently incubated with HCN2 (D1C6I) antibody (Cell Signaling Technology, Danvers, USA; Cat# 14957, RRID: AB_2798661). Actinin (Sigma–Aldrich, St. Louis, MO, USA; #A7811; RRID: AB_476766) was used as a loading control. Immunoreactive proteins were visualised by chemiluminescent detection (#WBLUF0, Immobilon Forte Western HRP substrate; Millipore Corporation, USA) and visualised using UVITEC Imaging Systems (Uvitec Limited, Cambridge, UK). Quantification was performed using threedimensional densitometry in Optiquant (Packard Instruments, RRID:SCR_016769). These arbitrary density levels were normalized to the appropriate loading controls and calculated to average control level to allow comparison of the protein amount between groups.

### Data analysis

Data are reported as the mean and standard error of the mean (SEM). Data with only two groups were analysed by unpaired t test or Mann–Whitney test according to data distribution. Normality of data distribution was evaluated by Shapiro–Wilk test. For more than 2 evaluated groups we used one-way ANOVA. P < 0.05 was considered statistically significant. The data were handled by GraphPad Prism 6 for Windows (Graph Pad Software, Inc., version 6.00; RRID: SCR_002798).

## Results

### Basic features of disease development

Diabetes mellitus in STZ animals was confirmed by increased blood glucose levels (Table 2). We found absolute body and heart weight reduction in STZ rats, but the relative heart weight was not changed. Presence of cardiac damage was suggested by a significant myosin-isoform shift (Table 2).

**Table 2:** Characteristics of experimental groups

	CON	STZ
*Weight outcomes (n)*	10	10
Body weight (g)	438 ± 15	289 ± 14*
Heart weight (mg)	1238 ± 83	840 ± 28*
Heart weight/body weight (mg/g)	2.8 ± 0.2	2.9 ± 0.1
*Blood glucose measurement (n)*	10	10
Casual glycaemia (mmol/l)	7.4 ± 0.5	20.4 ± 1.79*
*Cardiac mRNA expression (n)*	9	10
Myh6 (%, relative to controls)	100 ± 5	29 ± 4*
Myh7 (%, relative to controls)	100 ± 17	270 ± 16*

Mean ± SEM, *p < 0.05 versus CON

CON—controls, STZ—streptozotocin administered diabetic rats

### Electrocardiographic abnormalities

In STZ model, typical ECG alterations were observed (Table 3), including slower heart rate (about 20%, *P* < 0.05), prolonged QT and QTc intervals, wider QRS complex as well as prolonged T wave.

**Table 3:** ECG evaluation using II. lead

	CON	STZ
*ECG parameters (n)*	10	9
*Heart rate* (bpm)	349 ± 16	297 ± 8.9*
R –R (ms)	175 ± 8.2	203 ± 6.1*
P –wave (ms)	23 ± 1.5	23 ± 0.8
P-Q interval (ms)	49 ± 3.1	55 ± 2.8
QRS interval (ms)	16 ± 0.4	18 ± 0.7*
T- wave (ms)	49 ± 3.2	64 ± 2.6*
QT (ms)	64 ± 3.1	83 ± 2.5*
QTc (ms)	69 ± 3.2	82 ± 2.6*
P amplitude (mV)	0.09 ± 0.01	0.15 ± 0.01*
R amplitude (mV)	0.6 ± 0.05	0.9 ± 0.06*
T amplitude (mV)	0.14 ± 0.01	0.26 ± 0.03*

Mean ± SEM, *p < 0.05 versus CON

CON—controls, STZ—streptozotocin administered diabetic rats

### Downregulated Hcn2 and potassium currents regulating genes

**Fig. 1:**
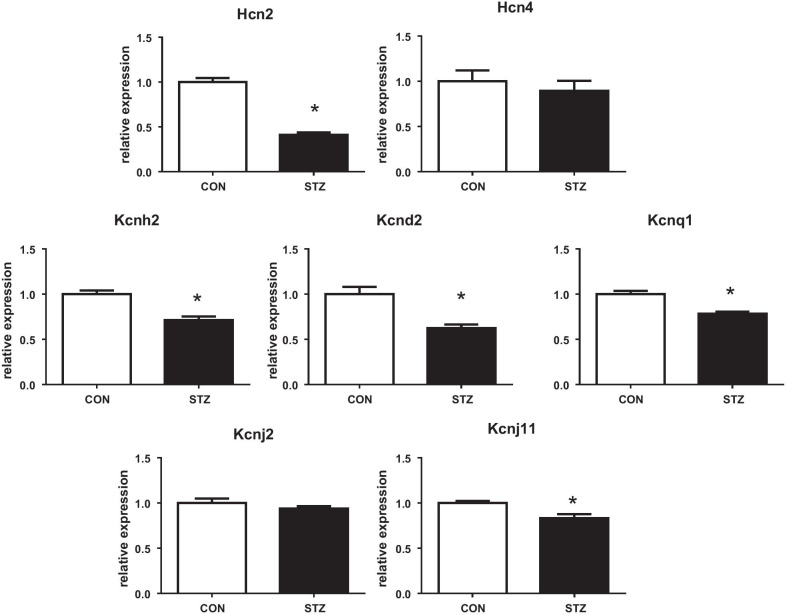
Relative cardiac mRNA expressions of Hcn channels and potassium channels (Kcnd2, Kcnh2, Kcnq1, Kcnj2, Kcnj11; for details see Table 1) in rat hearts. Groups labelling: CON, controls; STZ, streptozotocin administered diabetic rats; data are presented as mean ± SEM; n = 9–10 per group; *P < 0.05 versus CON

Interestingly, we found a downregulation of mRNA of Hcn2 channels responsible for *I*_f_ current exclusively in left ventricles of diabetic rats (by 60%; *P* < 0.05, Fig. 1). In contrast, the expression of Hcn4 channels remained stable. As we observed profound abnormalities of repolarization, we analysed the mRNA expression of key genes encoding respective potassium channels. Except of Kcnj2 (determining *I*_RK1_ current), all assessed potassium channels were downregulated in diabetic heart, namely: Kcnd2 (*I*_to1_), Kcnh2 (*I*_Kr_), Kcnq1 (*I*_Ks_) and Kcnj11 (*I*_KATP_; Fig. 1).

### Downregulated Hcn2 protein exclusively in STZ ventricles

**Fig. 2:**
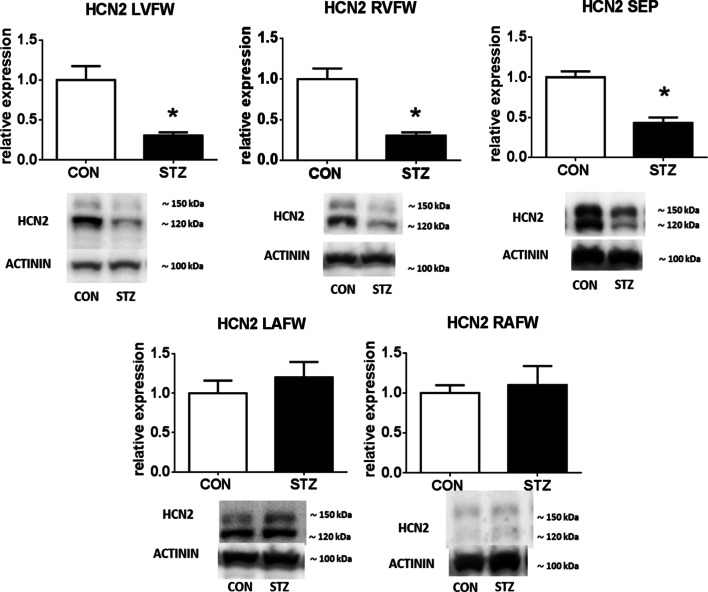
Western blot analysis of HCN2 channels in different regions of the heart of control and diabetic rats with representative immunoblots. Two bands are visible, likely corresponding to unglycosylated (displayed at ≈ 120 kDa) and N-glycosylated (displayed at ≈ 150 kDa) forms of the HCN2. Data are reported as relative cardiac protein expression of HCN2 channels in rat free wall of left ventricle (LVFW), right ventricle (RVFW), septum (SEP), left atrium (LAFW) and right atrium (RAFW). For groups labelling see Fig. 1. Data are presented as mean ± SEM; n = 10 per group; *P < 0.05 versus CON; full-length blots are presented in Additional file 1

Subsequently, we evaluated protein expression of HCN2 protein in the different regions of the rat heart (Fig. 2). Western blot analysis revealed downregulation of HCN2 protein, exclusively, in the diabetic rat ventricles (i. e. left and right ventricle 70%; *P* < 0.05 and septum 57%; *P* < 0.05). The expressions of HCN2 in left and right atria after 6 weeks of diabetes were unaltered as compared to controls.

### Unaltered expression of miR-1 and miR-133a

**Fig. 3:**
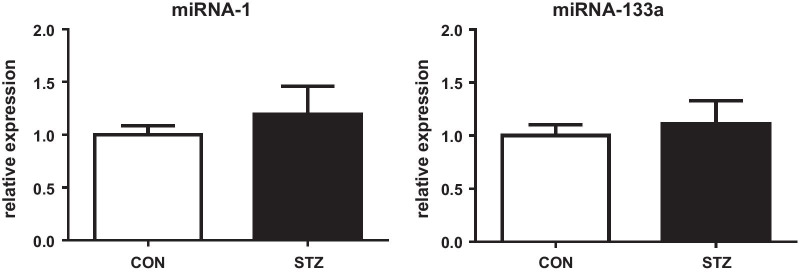
Relative cardiac microRNA (miR-1 and miR133a) expressions. Mean ± SEM; n = 9–10 per group; for labelling of groups see Fig. 1

To investigate one possible way of regulation of HCN2 expression, we determined short non-coding, single-stranded RNA molecules microRNAs (miRs) regulating transcriptional and post-transcriptional gene expression. We assessed two: miR-1-3p and miR-133a-3p predicted to target cardiac Hcn2 and Hcn4 channels. However, expression of analysed mature microRNAs was not changed (Fig. 3).

### Stable Hcn expressions under hyperglycaemia in partially differentiated H9c2 cell line

**Fig. 4:**
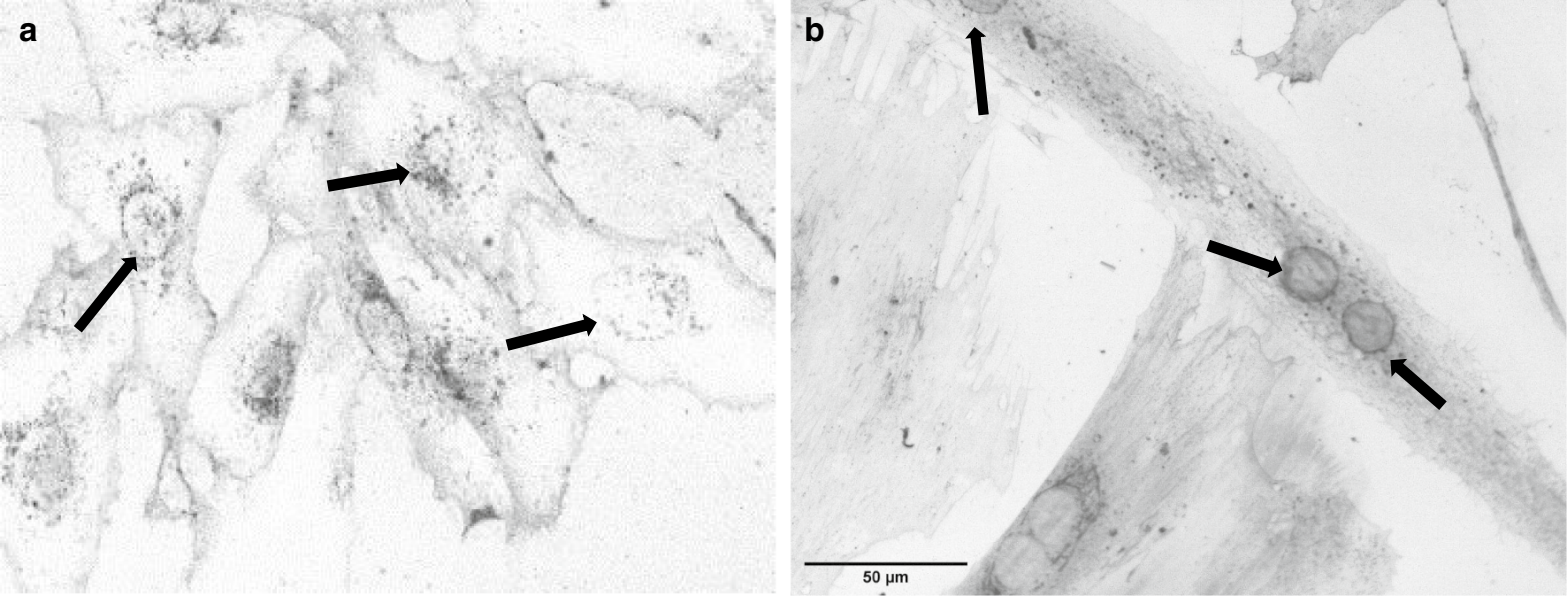
**a** Morphology of undifferentiated mononucleated and small spindle shaped myoblasts; **b** Decrease of glucose and serum led to the appearance of multinucleated long myotubes. Arrows indicate nucleus location within the cells; Greyscale: Wheat Germ Agglutinin (WGA) Alexa Fluor 488 Conjugate

The differentiation of cells was confirmed by elevated Tnnt2 mRNA expression (*P* < 0.05; Table 4) and morphology changes (Fig. 4). The differentiation enhanced expression of Hcn4 channels and glucose transporters Glut1 and Glut4 (Table 4). Still, these cells, even after differentiation, did not express genes typical for mature cardiomyocytes such as Myh6, Myh7, Tnni3, Kcnd2 and Ryr2 or their expression was only barely detectable (Cq value > 35). In order to investigate the impact of hyperglycaemia we determined mRNA expression of the genes of interest in H9c2 cell culture treated with high glucose, so individually examining one of the features of diabetes mellitus—hyperglycaemia, resp. PCR analysis did not reveal changes in expression of neither Hcn2 nor Hcn4 channels treated with high glucose (Table 4). We found Glut4 mRNA downregulation in hyperglycaemic conditions (Table 4).

**Table 4:** Relative mRNA expressions of the genes of interest in H9c2 cell line

	LG-DIF	HG-DIF	M-DIF	NON-DIF
*Cardiac gene expression (n)*	3	3	3	2
Hcn2 (%, relative to LG-DIF)	1.00 ± 0.02	1.07 ± 0.04	1.02 ± 0.03	1.00 ± 0.03
Hcn4 (%, relative to LG-DIF)	1.00 ± 0.05	0.94 ± 0.02	0.88 ± 0.04	0.51 ± 0.02*#§
Tnnt2 (%, relative to LG-DIF)	1.00 ± 0.02	0.83 ± 0.00*	0.84 ± 0.01*#	0.26 ± 0.01*#§
Glut1 (%, relative to LG-DIF)	1.00 ± 0.08	0.78 ± 0.04*	0.96 ± 0.05*	1.93 ± 0.18*#§
Glut4 (%, relative to LG-DIF)	1.00 ± 0.05	0.72 ± 0.03*	1.29 ± 0.06*#	0.41 ± 0.00*#§

Expression of Hcn channels (Hcn2 and Hcn4), cardiac marker Tnnt2 and glucose transporters (Glut1 and Glut4) in differentiated H9c2 cells; for details see Table 1) in H9c2 cell line. Mean ± SEM, n = 2–3 per group; *P < 0.05 vs LG-DIF, #*P* < 0.05 vs HG-DIF, §*P* < 0.05 vs M)

Groups labelling: LG-DIF—differentiated control cells treated with standard 5.5 mM glucose, HG-DIF—hyperglycaemia, differentiated cells treated with 33 mM glucose, M-DIF—mannitol, differentiated cells treated with 5.5 mM glucose and 27.5 mM of mannitol, NON-DIF – cells harvested before the differentiation

## Discussion

Experimental DM due to STZ administration in rats exhibits typical ECG changes which can be considered as common signs of developing diabetic cardiomyopathy [2, 20]. Although implying pro-arrhythmogenic potential, studies showed rather reduced risk of triggered ventricular dysrhythmias in STZ model [4, 5]. This is in line with certain evidence showing less tendency of diabetic hearts to ventricular arrhythmias regardless of pronounced risk of cardiovascular morbidity and mortality in diabetic patients [4, 6, 34] but it is contradictory to the impaired cardiac repolarization in DM [2, 35] suggesting effective compensatory mechanisms. The main finding of our study in this model is the downregulation of HCN2 channel exclusively in ventricular myocardium possibly contributing to regulation of electric stability of diabetic ventricles.

The cardiac downregulation of HCN channels under pathologic conditions is not an isolated finding, though related mostly to atria. HCN downregulation in sinus node is reported in aged heart [36] and heart failure and relates to sinus node dysfunction [37] and even atrial tachyarrhythmia [38]. Similarly, a decrease of HCN was found in the atria of metabolic murine model of mitochondrial dysfunction [19] and in sinoatrial node of Goto-Kakizaki type 2 diabetic rats [39]. Importantly, HCN downregulation was reported also in pacemaker cells [20], in sinoatrial node [20] and whole cardiac conduction system [40] of rats with type 1 diabetes mellitus induced by STZ manifesting in lower intrinsic heart rate, a lengthened sinoatrial conduction time and rate-corrected maximal sinoatrial node recovery time in vivo as well as a longer cycle length in vitro [20]. Also, knock out mouse models lacking Hcn2 or Hcn4 channels exhibit sinoatrial heart disturbances without reports of ventricular electric abnormalities [8, 9] suggesting different roles of HCN channels in ventricles. Moreover, Hofmann et al. (2012) reported that HCN2/HCN4 deficiency results in a less pronounced prolongation of ventricular repolarization and a strong attenuation of pro-arrhythmogenic potential in settings of triggered ventricular hypertrophy. In light of this, the decrease of Hcn2 gene expression in ventricles is a novel and engaging finding that might contribute to electrical stability of diabetic ventricles in spite of presence of prolonged ventricular repolarization.

We observed altered expression of particular potassium channels-related genes particularly those significantly contributing to observed abnormal repolarization (*i.e.* prolonged QT interval and increased T wave) such as Kcnh2 (gene encoding the Kv11.1 subunit of ERG channel; [3]) and Kcnq1 (gene encoding the Kv7.1 subunit of KvLQT1 channel [3]). However, repolarization abnormalities, presumably, mirror a complex orchestration of changes during development of diabetic heart damage [41]. As mentioned above, HCN channels might be an integral part of cardiac electric remodelling and might play a role in cardiac repolarization as the enhanced activity of HCN channels is reported to disrupt ventricular repolarization and lengthen QT interval and double HCN2/HCN4 knockout in the ventricles of the hypertrophic hearts results in attenuated action potential and QTc interval prolongation [13]. However, these ECG alterations were detected in animals with normal expression of K^+^ channels what is different from our model where Hcn2 downregulation is a part of gene reprogramming. Since ventricular repolarization is mediated by K^+^ channels, prolonged QTc interval in STZ rats is likely due to K^+^ channels downregulation [42] usually responsible for attenuation of ventricular repolarization reserve and consequent proarrhythmic risk [41]. The Hcn2 downregulation in our study may be viewed as a compensatory phenomenon, supporting repolarization reserve.

Regulation of cardiac HCN channels expression may be affected by microRNAs [43] regulating transcriptional and post-transcriptional gene expression. We focused on miR-1 and miR-133a as their dysregulation contributes to diabetic cardiomyopathy [44] and moreover, also influences HCN2 and HCN4 expressions [43]. However, alterations of HCN2 found in our study were not accompanied by altered levels of miR-1 nor miR-133a questioning their contribution to electric remodelling in ventricles of STZ rats and suggesting a different mechanism for HCN2 downregulation.

To investigate the particular role of hyperglycaemia for HCN channels expression, we used differentiated H9c2 cell line treated with high glucose concentration in the culture medium. H9c2 cells, as an in vitro model, have been used in research of HCN channels previously [25] though they do not exhibit apparent endogenous pacemaker currents. Excess of glucose did not result in HCN channels expression changes in H9c2 cells indicating that hyperglycaemia per se is not sufficient to downregulate HCN2. We found a decrease in Glut4 glucose transporter mRNA expression after high glucose treatment which reflects disrupted glucose metabolism in H9c2 cells, similar to diabetic cardiomyopathy [45]. However, H9c2 cells are not maturated cells and they do not spontaneously beat even after differentiation. As the mature cardiac myocytes are characterized by structural and functional entities involved in the generation and transmission of the action potential and the excitation–contraction coupling process, one would expect differences in genotype between H9c2 and mature cardiomyocytes. In fact, the specific organization of ion channels and transporters promoting action potential is key to the function of the cardiac myocytes [46]. It was shown that mechanical stress [47] and electrical stimulation [48] which are physiologically relevant for cardiomyocytes increase expression of ion channels. Therefore, using H9c2 cell line to study electrophysiology is associated with certain limitations. On the other hand, the cell line expresses particular ion channels typically observed in cardiomyocytes and some electrophysiological properties were documented suggesting H9c2 cells are potentially valuable surrogates for the investigation of ion channel regulation [49]. However, we did not detect any noticeable expression of transient outward current subunit Kcnd2 and ryanodine receptor which modulate the spontaneous beating rate of cardiomyocytes during development and therefore are crucial for cardiomyocyte function [50]. Consequently, our results underline the fact, that observed HCN2 downregulation in diabetes is highly specific for ventricular myocytes and it is not observed in either atrial tissue or H9c2 cells (Additional file 1).

## Conclusion

Conclusively, diabetic rat heart displays ECG alterations whose severity and possible progression into rhythm disturbances are possibly modulated also by decreased expression of HCN2 channels. This downregulation might balance the influence of altered expression of dominant repolarization-related potassium channels and, as it occurs exclusively in ventricular tissue in vivo, it may serve as a protective mechanism particularly against ventricular dysrhythmias. Whether it is crucially responsible for reduced susceptibility of diabetic heart to arrhythmias needs further clarification.

## Supplementary information

**Additional fle 1.** Full-length immunoblots of HCN2 channels in the different regions of the heart of control and diabetic rats.

## Data Availability

The datasets used and/or analysed during the current study are available from the corresponding author on reasonable request.

## Abbreviations

B2m: β2-Microglobulin
DM: Diabetes mellitus
DMEM: Dulbecco's modified eagle's medium
EGG: Electrocardiography
FBS: Fetal bovine serum
Hcn2: Hyperpolarization activated cyclic nucleotide gated potassium and sodium channel 2
Hcn4: Hyperpolarization activated cyclic nucleotide gated potassium and sodium channel 4
HG-DIF: Hyperglycaemia, differentiated cells
Hprt1: Hypoxanthine phosphoribosyltransferase 1
Kcnd2: Potassium voltage-gated channel subfamily D member 2
Kcnh2: Potassium voltage-gated channel subfamily H member 2
Kcnj11: Potassium inwardly rectifying channel subfamily J member 11
Kcnj2: Potassium inwardly rectifying channel subfamily J member 2;
Kcnq1: Potassium voltage-gated channel subfamily Q member 1
LG-DIF: Differentiated control cells treated with standard 5.5 mM glucose
M-DIF: Differentiated control cells treated with mannitol
miR: MicroRNA
Myh6: Myosin heavy chain 6 (α‐myosin heavy chain)
Myh7: Myosin heavy chain 7 (β‐myosin heavy chain)
NON-DIF: Cells harvested before the differentiation
RT-qPCR: Real-time quantitative polymerase chain reaction
Ryr2: Ryanodine receptor; 2
Sdha: Succinate dehydrogenase complex flavoprotein subunit A
Slc2a1: Solute carrier family 2 member 1 (alias Glut1)
Slc2a4: Solute carrier family 2 member 4 (alias Glut4)
STZ: Streptozotocin
Tnnt2: Troponin T2, cardiac type
Tnni3: Troponin I3, cardiac type;
WGA: Wheat germ agglutinin
