# Supplementary material for: Isolated downregulation of HCN2 in ventricles of rats with streptozotocin-induced diabetic cardiomyopathy
Source: BMC Cardiovasc Disord. 2021 Mar 2;21:118. doi: 10.1186/s12872-021-01929-3 (PMC7927235; doi:10.1186/s12872-021-01929-3)
Supplement: Supplementary file 1 — Additional fle 1. Full-length immunoblots of HCN2 channels in the different regions of the heart of control and diabetic rats. [file 12872_2021_1929_MOESM1_ESM.docx]

**Supplementary Data for Original Immunoblots of Fig.2 in Manuscript:**

Isolated Downregulation of HCN2 in Ventricles of Rats with Streptozotocin-Induced Diabetic Cardiomyopathy

AUTHORS

Katarina Hadova^1^, Eva Kralova^1^, Gabriel Doka^1^, Lenka Bies Pivackova^1^, Zuzana Kmecova^1^, Peter Krenek^1^, Jan Klimas^1*^

AFFILIATIONS

^1^Department of Pharmacology and Toxicology, Faculty of Pharmacy, Comenius University in Bratislava, Slovakia

***Corresponding author:** prof. Jan Klimas (email: [jan.klimas@uniba.sk](mailto:jan.klimas@uniba.sk); ORCID: <https://orcid.org/0000-0002-6845-2105>), Department of Pharmacology and Toxicology, Faculty of Pharmacy, Comenius University in Bratislava, Odbojarov 10, 832 32 Bratislava, Slovakia

Suplementary Fig.1: Full-length immunoblots of HCN2 channels in the different regions of the heart of control and diabetic rats. Two bands are visible, likely corresponding to unglycosylated (displayed at ≈120 kDa) and N-glycosylated (displayed at ≈150 kDa) forms of the HCN2. (Fig.2 in manuscript). Abbreviations: left ventricle (LVFW), right ventricle (RVFW), septum (SEP), left atrium (LAFW) and right atrium (RAFW), CON, controls; STZ, streptozotocin administered diabetic rats

Original HCN2 LVFW – in the cropped Fig.2 in manuscript upper LINE 7 and LINE 8 (indicated by the frame) are represented





HCN2

HCN2

~ 120 kDa

~ 150 kDa

~ 120 kDa

~ 150 kDa

STZ

CON

STZ

CON

STZ

CON

STZ

CON

STZ

CON

CON

STZ

STZ

STZ

STZ

STZ

CON

CON

CON

CON

Original Actinin LVFW - in the cropped Fig.2 in manuscript upper LINE 7 and LINE 8 (indicated by the frame) are represented




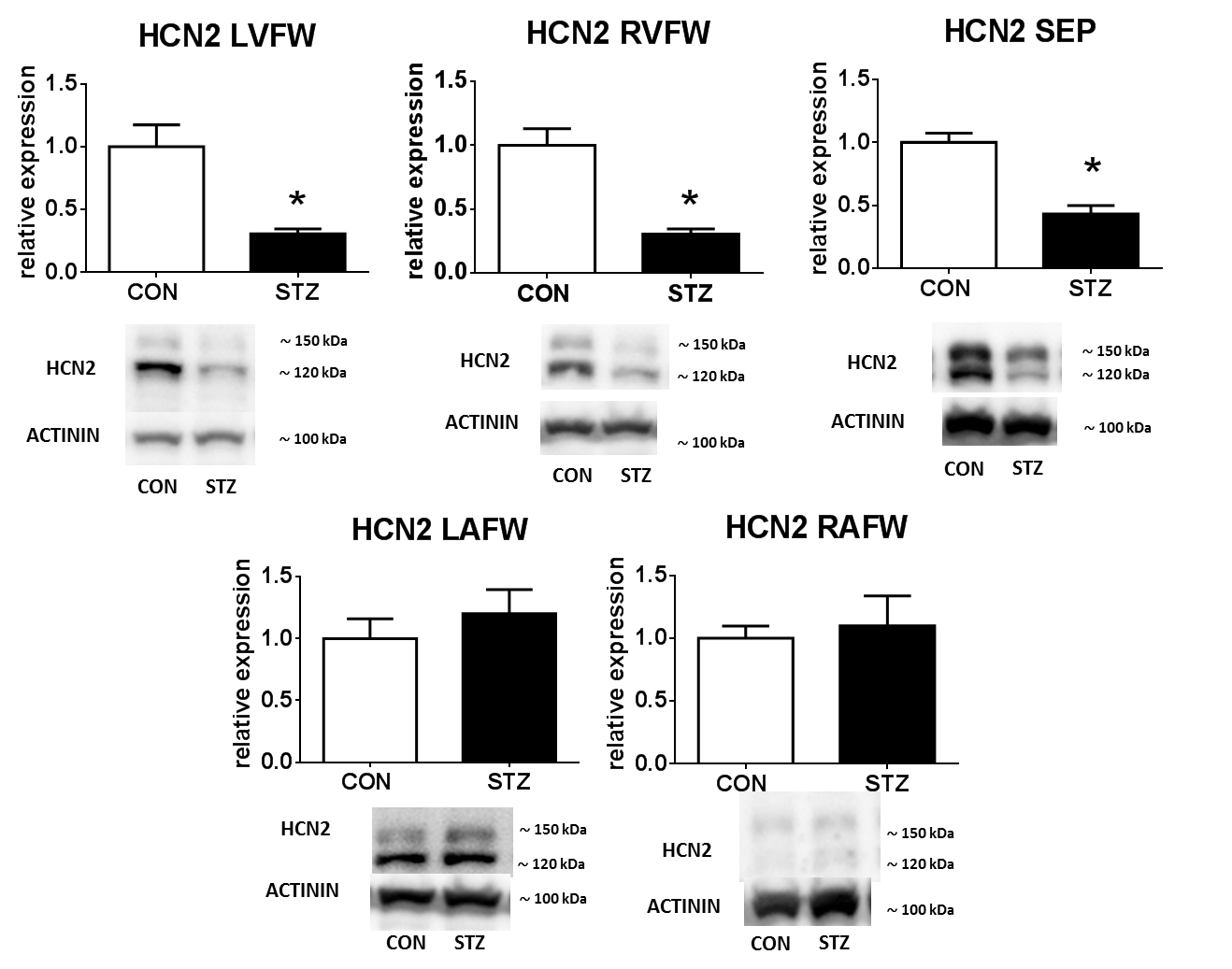


ACTININ

ACTININ

~ 100 kDa

~ 100 kDa

CON

CON

CON

CON

CON

STZ

STZ

STZ

STZ

STZ

CON

CON

CON

CON

CON

STZ

STZ

STZ

STZ

STZ

Original HCN2 RVFW – in the cropped Fig.2 in manuscript upper LINE 7 and LINE 8 (indicated by the frame) are represented





HCN2

HCN2

~ 120 kDa

~ 150 kDa

~ 120 kDa

~ 150 kDa

CON

CON

CON

CON

CON

STZ

STZ

STZ

STZ

STZ

STZ

STZ

STZ

STZ

STZ

CON

CON

CON

CON

CON

Original Actinin RVFW – in the cropped Fig.2 in manuscript upper LINE 7 and LINE 8 (indicated by the frame) are represented




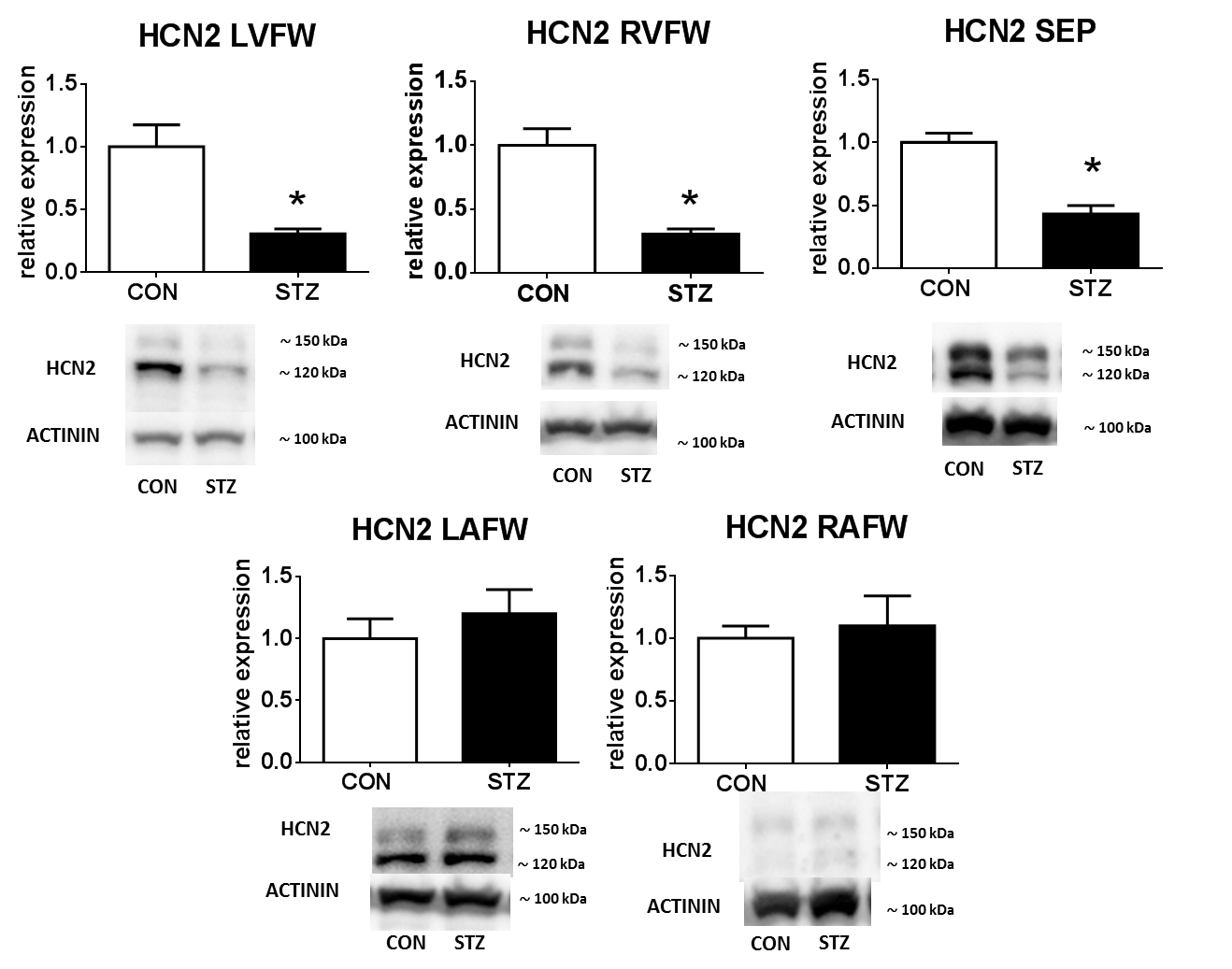


ACTININ

ACTININ

~ 100 kDa

~ 100 kDa

STZ

STZ

STZ

STZ

STZ

CON

CON

CON

CON

CON

CON

CON

CON

CON

CON

STZ

STZ

STZ

STZ

STZ

Original HCN2 SEP – in the cropped Fig.2 in manuscript upper LINE 7 and LINE 8 (indicated by the frame) are represented





HCN2

HCN2

~ 150 kDa

~ 120 kDa

~ 150 kDa

~ 120 kDa

STZ

STZ

STZ

STZ

STZ

CON

CON

CON

CON

CON

STZ

STZ

STZ

STZ

STZ

CON

CON

CON

CON

CON

Original Actinin SEP – in the cropped Fig.2 in manuscript upper LINE 7 and LINE 8 (indicated by the frame) are represented




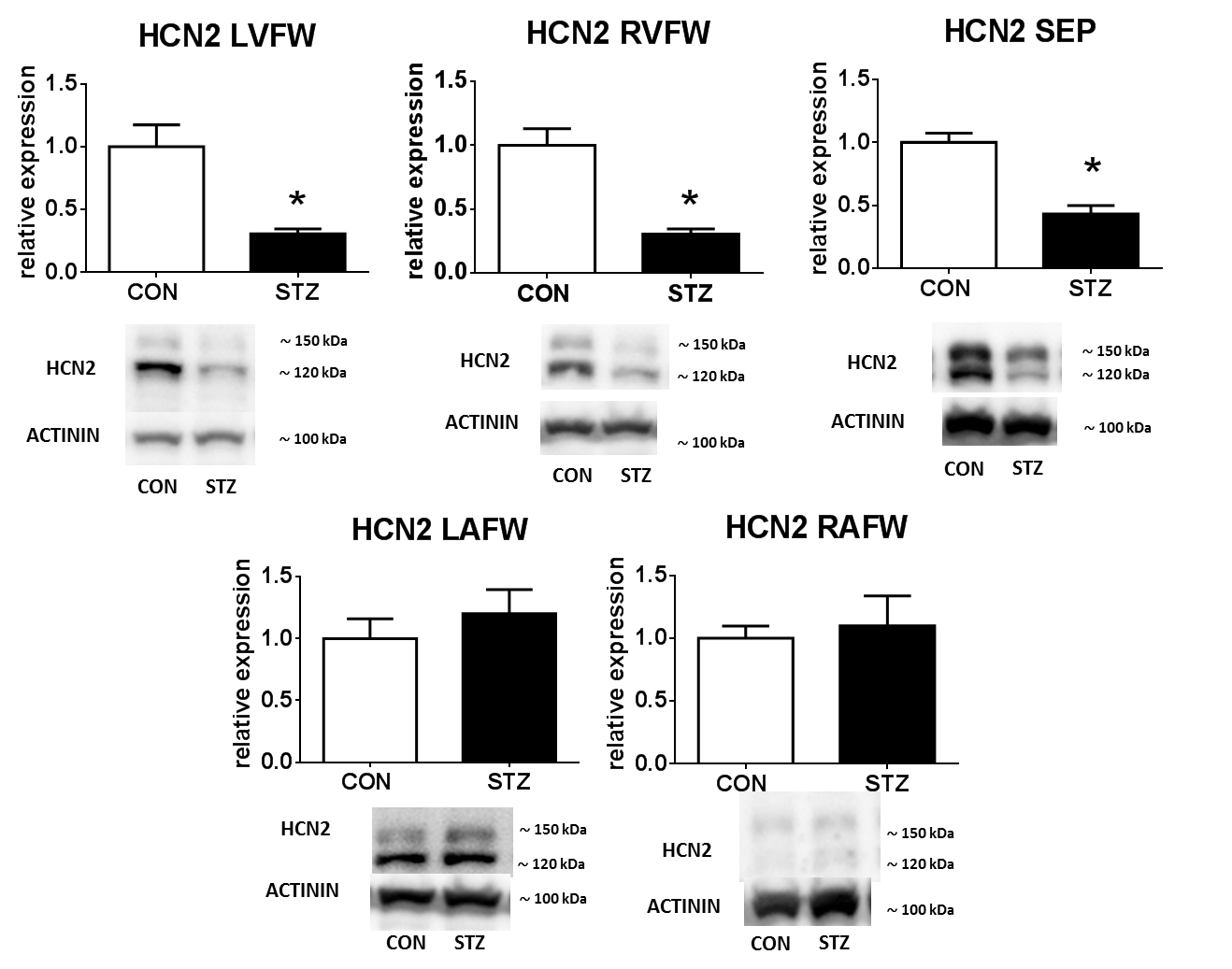


ACTININ

ACTININ

~ 100 kDa

~ 100 kDa

STZ

STZ

STZ

STZ

STZ

CON

CON

CON

CON

CON

STZ

STZ

STZ

STZ

STZ

CON

CON

CON

CON

CON

Original HCN2 LA – in the cropped Fig.2 in manuscript upper LINE 3 and LINE 4 (indicated by the frame) are represented





HCN2

HCN2

~ 120 kDa

~ 150 kDa

~ 120 kDa

~ 150 kDa

STZ

STZ

STZ

STZ

STZ

CON

CON

CON

CON

CON

STZ

STZ

STZ

STZ

STZ

CON

CON

CON

CON

CON

Original Actinin LA – in the cropped Fig.2 in manuscript upper LINE 3 and LINE 4 (indicated by the frame) are represented




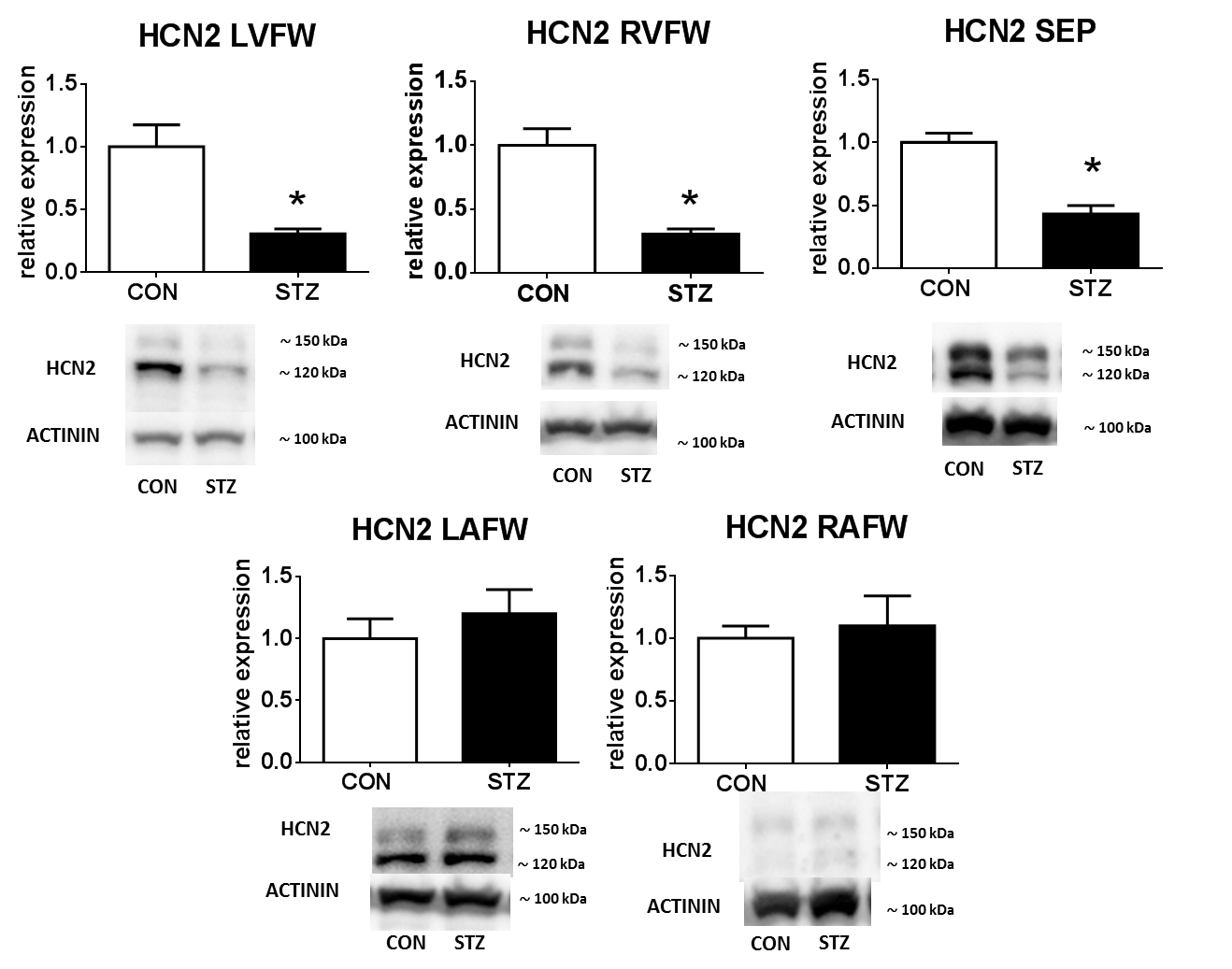


ACTININ

ACTININ

~ 100 kDa

~ 100 kDa

STZ

STZ

STZ

STZ

STZ

CON

CON

CON

CON

CON

STZ

STZ

STZ

STZ

STZ

CON

CON

CON

CON

CON

Original HCN2 RA – in the cropped Fig.2 in manuscript upper LINE 7 and LINE 8 (indicated by the frame) are represented





HCN2

HCN2

~ 120 kDa

~ 150 kDa

~ 120 kDa

~ 150 kDa

CON

CON

CON

CON

CON

STZ

STZ

STZ

STZ

STZ

STZ

STZ

STZ

STZ

STZ

CON

CON

CON

CON

CON

Original actinin RA – in the cropped Fig.2 in manuscript upper LINE 7 and LINE 8 (indicated by the frame) are represented




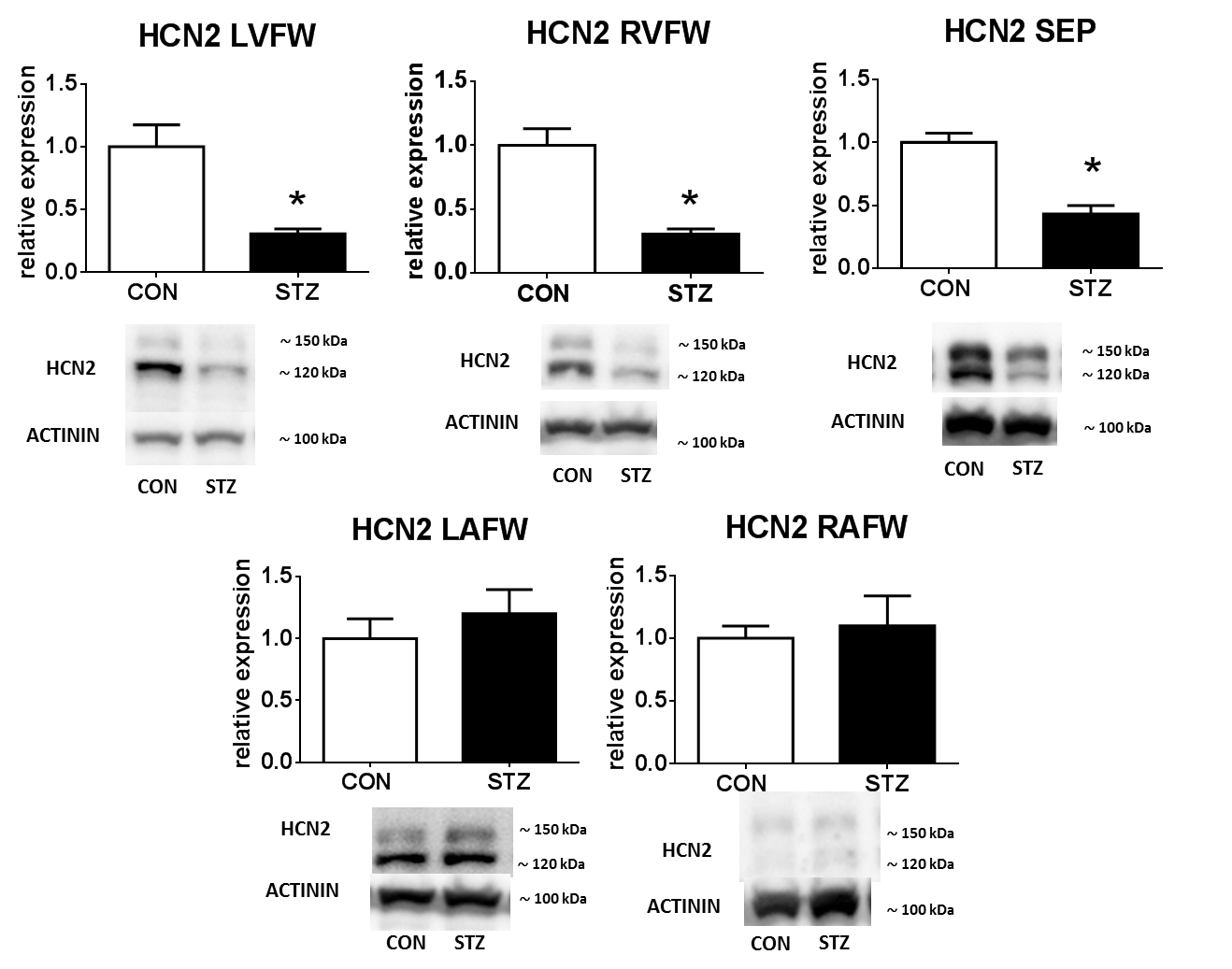


ACTININ

ACTININ

~ 100 kDa

~ 100 kDa

CON

CON

CON

CON

CON

STZ

STZ

STZ

STZ

STZ

STZ

STZ

STZ

STZ

STZ

CON

CON

CON

CON

CON
